# Response to: ‘Comment on: ‘Effect of trabeculectomy on the rate of progression of visual field damage”

**DOI:** 10.1038/s41433-023-02876-3

**Published:** 2023-12-22

**Authors:** Susanna Friederike Koenig, Giovanni Montesano, Clarissa Ern Hui Fang, David Paul Crabb, Hari Jayaram, Jonathan Clarke

**Affiliations:** 1 Moorfields Eye Hospital NHS Foundation Trust, 162 City Road, EC1V 2PD London, UK; 2 Universitaetsaugenklinik Ulm, Prittwitzstrasse 43, D – 89075 Ulm, Deutschland Germany; 3 Optometry and Visual Sciences, City, University of London, London, UK; 4 NIHR Biomedical Research Centre of Ophthalmology, Moorfields Eye Hospital and UCL Institute of Ophthalmology, London, UK

**Keywords:** Outcomes research, Optic nerve diseases

## To the Editor:

We thank the authors of the letter [1] for their interest in our work and the editor for the opportunity to respond.

As mentioned in the letter, and in our paper [2], the development of media opacity due to cataract can reduce visual field (VF) sensitivity. It is also documented that glaucoma surgery can accelerate the formation of cataract [3]. This effect would produce a worsening of global VF indices, such as Mean Deviation (MD), counteracting the stabilising effect of surgery. However, as the authors of the letter correctly suggest, cataract surgery can improve MD, contributing to a less negative rate of progression in the post-operative period.

**Fig. 1:**
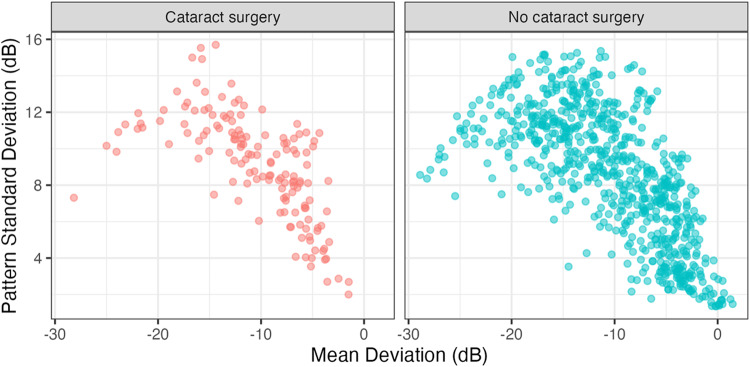
Distribution of pairs of Mean Deviation – Pattern Standard Deviation values in our cohort. The curvilinear relationship is evident in both groups of patients.

We should clarify that our main analysis did not use MD, but rather pointwise sensitivity. This allowed us to avoid biases from the perimetric measurement floor (Fig. 1 in the original paper). This analysis would not be possible with MD.

The authors of the letter suggested using Pattern Standard Deviation (PSD) to assess progression while minimising the effect of cataract surgery, perhaps misinterpreting the results of one of their references. Koucheki et al. [4] showed that changes in PSD after cataract surgery were mainly due to the increase in sensitivity in healthier locations (i.e. exhibiting changes independent of glaucoma progression). Moreover, PSD is inadequate to track progression, because of its nonlinear relationship with glaucoma damage (both healthy and very advanced fields would have low PSD [5]). This was also the case for our cohort (see Fig. 1). Therefore, a change in PSD cannot be interpreted, in isolation, as indicative of either worsening or improvement of the VF. Moreover, the median MD of our patients at surgery was −10.84 dB. This means that our metric of choice would need to track progression into the advanced stages, making measurements focused on the pattern of damage (pattern deviation or PSD) inappropriate.

However, to reassure the authors of the letter, we have performed our analysis after excluding patients who received cataract surgery. The results are largely similar to those obtained with the full dataset. The mean rate of progression before surgery was −0.93 [−1.23, −0.64] dB/year (Mean [95% Credible Intervals]) and it was slowed down by 0.61 [0.20, 1.03] dB/year (*p*_d_  = 0.0046).
